# A Memorable Christmas Day

**Published:** 1916-12-30

**Authors:** 


### The Hospital

The Workers' Newspaper of Administrative Medicine and Institutional Life, Administration, National Insurance and Health.

No. 1595, Vol. LXI. SATURDAY, DECEMBER 30, 1916. PRICE ONE PENNY

### A MEMORABLE CHRISTMAS DAY.

When the authorities of Guy's Hospital, more than a quarter of a century ago, began to make Christmas Day memorable in that hospital by turning each ward into a bower of Christmas beauty, owing to the infinite skill and wonderful taste displayed in its decoration, and when the staff and students, from the highest to the lowest, devoted most of Christmas Day, in the spirit of Christ, to brighten and cheer the sick in the wards, and to make the day one of innocent pleasure and gladness to the children who had been patients at the hospital during the preceding year, the whole atmosphere of the hospital was changed for the better. At first there were critics who condemned the new departure and clung to the undecorated ward and the absence of all signs of Christmas, as a sort of faith-inspiring protest, against inhygienic practices which carried a dash of paganism with them. Opposed to these views were those of the managers of a few hospitals, up and down the country, who had for many years previous to 1890 made Christmas Day a hospital festival for the patients. We have often described Christmas Day at Guy's Hospital, have upheld the principles on which it rests, and commended to all thoughtful people the privilege and advantage which are open to all who make a practice of visiting the patients in the wards of some hospital on Christmas Day. A life's experience has taught us that there is no place on Christmas Day like a hospital. This all will agree who habitually practise personal service in the days of health in the cause of the sick, as an act of praise to God for the blessings they enjoy. As the years have passed, an increasing number of earnest people of both sexes have followed our advice, and to them Christmas Day has proved an unfailing inspiration and blessing.

When King Edward's Hospital Fund was founded King George and Queen Mary set an example to every family in the land by sending an annual subscription to the Fund, not only for themselves, but for each of their children individually. On Christmas Day this year, for the first time, King George and Queen Mary spent some hours in King George Hospital, and, what is more, they were accompanied by all their children who were in London, each of whom took a floor and did His or Her best to cheer the patients, and enter into the spirit of Christmas Day with them. No one has been more consistent than His Majesty the King in devoting, deliberately, every year, a portion of his time to personal service in the cause of the sick. Queen Mary, notably since the war, has devoted more and more time to personal service, and we welcome the public advent of their children to the wards of a great hospital on Christmas Day. We hope they may continue the practice from this time forward as the years pass. Queen Alexandra spent some time on Christmas Day with the patients in her own hospital, the Alexandra Hospital for Children, where she did good and welcome service to many a sorely stricken child. Their Majesties and their children could have done nothing more inspiriting to hospital workers, or more helpful to the uplifting and permanent benefit of our hospitals, than the personal service they spontaneously rendered to the sick on Christmas Day 1916. We are encouraged to hope that Their Majesties' example will be followed throughout the United Kingdom by the majority, at any rate, of those who lead public opinion in that portion of the country where they habitually live. With such action our hospitals will not be only great palaces of pain and cure. For then the Hospitals will tend to quicken the spiritual life of the people in these islands, to an extent the value and importance of which cannot be overestimated. God Save Your Majesties!

